# From Lung to Heart: Unusual Case of Pulmonary Myxoid Sarcoma

**DOI:** 10.1016/j.jaccas.2024.102919

**Published:** 2024-12-18

**Authors:** Norma Nicole Gamarra-Valverde, Fernando Untiveros-Mayorga, Jose Somocurcio-Peralta, Sebastian Reyes-Villanes, Javier Torres-Valencia

**Affiliations:** a Alberto Hurtado Faculty of Human Medicine, Universidad Peruana Cayetano Heredia, Lima, Peru; b Department of Cardiology, Hospital Nacional Edgardo Rebagliati Martins, Lima, Peru; c Department of Anatomical Pathology, Hospital Nacional Edgardo Rebagliati Martins, Lima, Peru

**Keywords:** cancer, echocardiography, imaging

## Abstract

A 77-year-old man with multiple comorbidities presented with cough, dyspnea and nonspecific malaise. Chest computed tomography revealed a mass in the right lower lobe of the lung, along with an associated hypodense lesion in the left atrium. Echocardiography showed a mobile mass in the left atrium, initially suspected to be a thrombus. Transesophageal echocardiography and contrast-enhanced computed tomography demonstrated a 7.5 × 4 × 5-cm tumor originating from the right inferior pulmonary vein, extending into the left atrium and ventricle, causing severe mitral stenosis. Lung biopsy confirmed primary pulmonary myxoid sarcoma. This rare case highlights the importance of comprehensive cardiac imaging in differentiating intracardiac masses and the challenges in diagnosing uncommon malignancies with atypical presentations.

## Graphical Abstract

**Figure:**
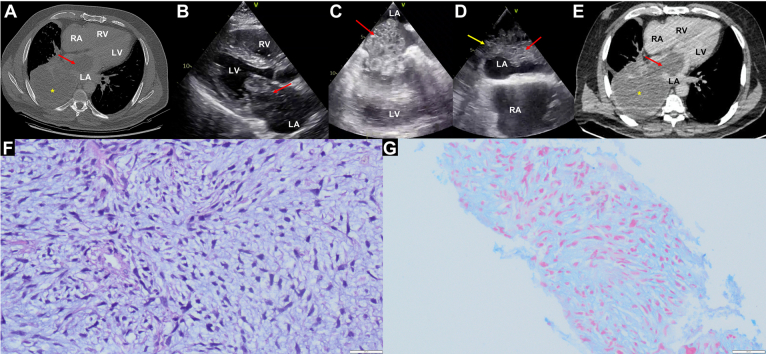

A 77-year-old man with a history of aortocoronary bypass surgery 25 years ago, unspecified interstitial lung disease, type 2 diabetes mellitus, chronic smoking, arterial hypertension, peripheral arterial insufficiency, and permanent atrial fibrillation on regular warfarin treatment presented to the emergency department. He had been experiencing recurrent episodes of cough with clear expectoration for 3 months, associated with dyspnea on mild exertion, general malaise, and a sensation of unquantified fever.

**Take-Home Messages**

• Transesophageal echocardiography is crucial for detailed cardiac imaging, providing comprehensive assessment of cardiac masses and guiding clinical decision-making in rare cases such as primary pulmonary myxoid sarcoma.
• Primary pulmonary myxoid sarcoma, though exceptionally rare, can present with atypical cardiac manifestations, underscoring the need for a high index of suspicion and thorough diagnostic workup in cases of unusual intracardiac masses.

Initial physical examination revealed the patient was afebrile, with blood pressure 160/80 mm Hg, heart rate 100 beats/min, respiratory rate 18 breaths/min, and oxygen saturation 93% on 28% FiO_2_. Cardiac auscultation revealed irregular heart sounds without murmurs. Lung auscultation showed decreased vesicular breath sounds at the right lung base, prolonged expiratory time, and scattered crackles at both lung bases.

Laboratory analyses reported troponin T of 0.0057 ng/L, D-dimer of 0.43 μ/mL, prothrombin time of 21.6 seconds, international normalized ratio of 1.9, and N-terminal pro–B-type natriuretic peptide of 848.1 pg/mL. The electrocardiogram showed atrial fibrillation with controlled ventricular response and poor R-wave progression in precordial leads, without signs of acute ischemia.

Given the physical examination findings, a noncontrast chest computed tomography (CT) was performed. It revealed an extensive mass in the right lower lobe extending to the ipsilateral bronchus and mediastinal lymphadenopathy, along with hypodensity in the left atrium and minimal pleural effusion (Figure 1A, Video 1).

**Figure 1:**
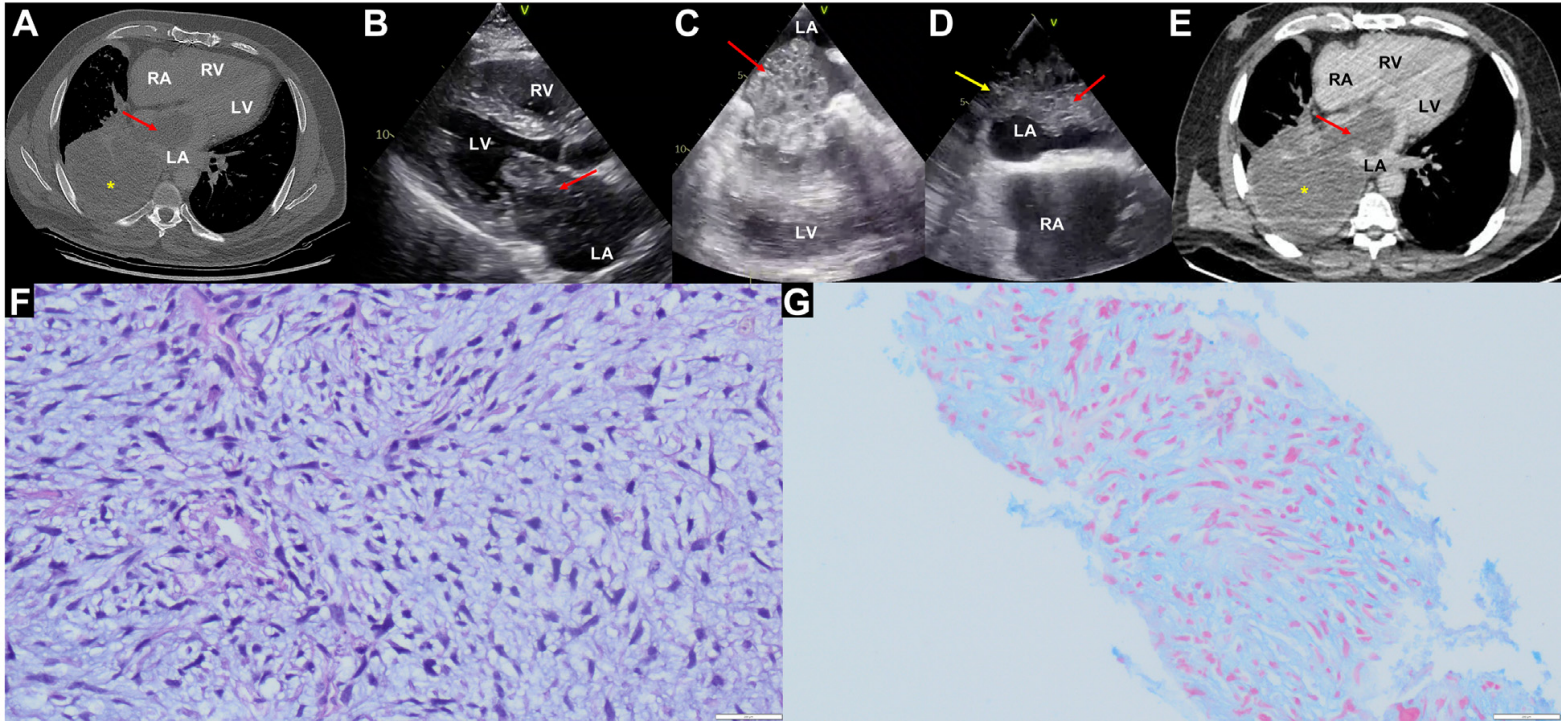
Multimodal Imaging of Pulmonary Myxoid Sarcoma With Right Lower Pulmonary Vein Extension to LA (A) Noncontrast computed tomography scan reveals a solid tissue mass in the right lower lobe (yellow asterisk), concurrent with a hypodense lesion in the left atrium (LA). (B) Transthoracic echocardiography: parasternal long-axis view shows a large left atrial mass protruding through the mitral valve (red arrow). (C) Transesophageal echocardiography: 4-chamber view revealing a large multilocular left atrial mass (red arrow) protruding through the mitral valve. The mass was associated with severe mitral flow obstruction, with a mean gradient of 12 mm Hg. (D) Bicaval view demonstrates that the mass (red arrow) is not attached to the atrial septum, but rather originates from the right lower pulmonary vein (yellow arrow). (E) Contrast-enhanced chest computed tomography demonstrates heterogeneous enhancement of both the mass located in the right lower lobe (yellow asterisk) and the lesion within the left atrium (red arrow.) (F) Hematoxylin and eosin–stained micrograph at 40× magnification demonstrates a sarcomatous neoplasm comprising hyperchromatic fusiform and stellate cells immersed in an abundant myxoid stroma. (G) Alcian blue histochemical staining accentuates the interstitial mucinous component, confirming the presence of acidic mucopolysaccharides. LV = left ventricle; RA = right atrium; RV = right ventricle.

A transthoracic echocardiography was subsequently performed, revealing a mobile mass with an irregular and heterogeneous surface in the left atrium, protruding into the left ventricle during diastole (Figure 1B, Videos 2 and 3). With the initial diagnostic suspicion of a malignant lung neoplasm complicated by a left atrial mass suggestive of thrombus, further investigation with transesophageal echocardiography and contrast-enhanced chest CT was pursued.

Transesophageal echocardiography showed a mobile heterogeneous tumor in the left atrium measuring 7.5 × 4 × 5 cm (Figure 1C, Video 4), originating from the right inferior pulmonary vein (Figure 1D, Video 5) and protruding into the left ventricle (Figure 1C), causing severe mitral outflow obstruction (mean gradient: 12 mm Hg). The left atrial appendage was free of thrombi. CT scan confirmed these findings and excluded the possibility of thrombus (Figure 1E).

A lung biopsy was obtained via fine-needle aspiration. Two days later, a multidisciplinary medical board convened. Given the high surgical risk and apparent tumor unresectability, they initially recommended a palliative approach. However, without biopsy results, specific treatment options could not be proposed. Despite hemodynamic stability, the patient suffered sudden cardiac death the following day.

In the following days, pathological analysis revealed mesenchymal tumor tissue with stellate, pleomorphic cells, myxoid matrix, and extensive necrosis with nuclear atypia (Figure 1F). Immunohistochemical analysis with Alcian blue staining was positive for myxoid material (Figure 1G), confirming the diagnosis of pulmonary myxoid sarcoma.

Primary pulmonary myxoid sarcoma is an extremely rare lung sarcoma. It is most frequently located in the right lower lobe and commonly infiltrates bronchial tissues, with a tendency toward endobronchial growth.^1^ This case report is the first to demonstrate extension into the left atrium through a pulmonary vein.

## Funding Support and Author Disclosures

The authors have reported that they have no relationships relevant to the contents of this paper to disclose.

## Appendix

Video 1 Noncontrast chest computed tomography reveals a solid tissue mass in the right lower lobe, concurrent with a hypodense lesion in the left atrium.

Video 2 Transthoracic echocardiography: parasternal long-axis view shows a large left atrial mass protruding through the mitral valve.

Video 3 Transthoracic echocardiography: color flow Doppler imaging in the parasternal long-axis view.

Video 4 Transesophageal echocardiography: 4-chamber view revealing a large multilocular left atrial mass protruding through the mitral valve.

Video 5 Transesophageal echocardiography: bicaval view demonstrates that the mass is not attached to the atrial septum, but rather originates from the right lower pulmonary vein.
